# Perspective Taking and Avatar-Self Merging

**DOI:** 10.3389/fpsyg.2022.714464

**Published:** 2022-03-18

**Authors:** Jochen Müsseler, Sophia von Salm-Hoogstraeten, Christian Böffel

**Affiliations:** Institute of Psychology, Work and Engineering Psychology, RWTH Aachen University, Aachen, Germany

**Keywords:** perspective taking, minimal self, avatar-self merging, Theory of Event Coding, avatar embodiment, spatial compatibility, ownership, agency

## Abstract

Today, avatars often represent users in digital worlds such as in video games or workplace applications. Avatars embody the user and perform their actions in these artificial environments. As a result, users sometimes develop the feeling that their self merges with their avatar. The user realizes that they are the avatar, but the avatar is also the user—meaning that avatar’s appearance, character, and actions also affect their self. In the present paper, we first introduce the event-coding approach of the self and then argue based on the reviewed literature on human-avatar interaction that a self-controlled avatar can lead to avatar-self merging: the user sets their own goals in the virtual environment, plans and executes the avatar’s actions, and compares the predicted with the actual motion outcomes of the avatar. This makes the user feel body ownership and agency over the avatar’s action. Following the event-coding account, avatar-self merging should not be seen as an all-or-nothing process, but rather as a continuous process to which various factors contribute, including successfully taking the perspective of the avatar. Against this background, we discuss affective, cognitive, and visuo-spatial perspective taking of the avatar. As evidence for avatar-self merging, we present findings showing that when users take the avatar’s perspective, they can show spontaneous behavioral tendencies that run counter to their own.

## Introduction

Originally, the term avatar referred to a deity of Indian mythology who descended to earth in a human appearance with the aim to enable mankind new insights, self-discoveries, and self-realizations. Nowadays, this term is transferred to virtual environments with abstract 2D outlines of individuals (e.g., a gravatar, Wolf and Henley, 2017) and 3D animated artificial characters (e.g., as illustrated in the movie “Avatar” by James Cameron, 2009). They are understood to either represent a completely independent artificial character or to act in place of a user in a virtual environment (Pan and Hamilton, 2018). In the present context, we refer to the term avatar in the latter sense. An avatar is understood as a (social) tool, as an extended “arm” of the user in video games and—increasingly also—in workplace applications. It enables the user to realize own intentions and goals in the virtual environment.

After intensive training and engagement with such an avatar, after navigating and interacting with it in the virtual environment, some users develop the feeling that they are integrating the avatar into their selves. They may even get the feeling of becoming one with it—a process we refer to as avatar-self merging (Böffel, 2021).^1^ We prefer this term as it captures the interactive influences of avatar and user. In gaming and virtual reality, the user realizes that they are the avatar, but the avatar is also them—meaning that its appearance, character, and actions affect also their self (Böffel, 2021). The avatar also does not replace only body parts, as various body-ownership illusions (e.g., Kilteni et al., 2015) and some prosthetic studies (e.g., Bekrater-Bodmann, 2020) suggest. For instance, arm amputees often report that their tool, the prosthetic arm, becomes a part of themselves after a period of training. We will argue that avatar-self merging goes beyond this because it emphasizes the interactive social component between user and avatar that pure ownership of body parts lacks.

In this paper, we synthesize existing studies and theories surrounding the user-avatar interaction and argue that controlling an avatar and taking its perspective is best described by the concept of avatar-self merging. We examine the conditions that facilitate but also constrain avatar-self merging. Before we do that, we need to clarify what the self is about and consider a prerequisite of successful avatar-self merging, namely, to successfully take the perspective of the (virtual) character.

## The Enrichment of the Self Through an Avatar: Avatar-Self Merging

Scientifically, two components are associated with the concept of the self: the minimal self and the narrative self (e.g., Gallagher, 2000). The minimal self is considered as the experience of our self in the here and now. Like other authors (e.g., Gallese and Sinigaglia, 2010; Hommel, 2018, 2021), we understand it as action-oriented, in the sense that it arises from our sensorimotor interactions with the environment. In contrast, the narrative self reflects our life experiences, which—among other events—contribute to our personal identity. It is assumed to need memory and language to be established.

Since the present context is primarily concerned with the sensorimotor interactions of users and their avatars in the virtual environment, we focus on the minimal self. More specifically, the interactions are assumed to give rise to the experiences of perceived body ownership and perceived agency, which in turn are seen as the constituting elements of the minimal self (see also Verschoor and Hommel, 2017). Perceived body ownership is understood as a person’s impression that their body belongs to them and is distinct from their environment. Healthy persons usually feel their hand belongs to them, but they may also perceive a rubber hand in front of them as part of their body if that rubber hand is oriented like their hand and stroked simultaneously with it (so-called rubber-hand illusion, Botvinick and Cohen, 1998; Costantini and Haggard, 2007).

Perceived agency refers to the impression of being the originator of an action and of controlling events in the environment with this action. This impression of being an agent arises when we lift a beverage with our hand, for example, but also when this is done indirectly with a mechanic gripping tool. In the latter case, the cognitive and motor performances (force, movement distance, etc.) can be completely different; nevertheless, we attribute the lifting action to us (e.g., Sutter et al., 2013).

Perceived ownership and perceived agency are seen to be intimately linked, modulated by each other (de Haan and de Bruin, 2010), and influenced by the same manipulations (Ma et al., 2019, 2021). Thus, it is not completely clear what separate contributions both concepts make to the minimal self. A further problem is that they are often gathered with subjective questionnaires, which are known to be prone to errors and biases. This has led to the concept of the minimal self being burdened with a certain degree of fuzziness.

Last but not least, there was a lack of ideas about how to conceive the representation of the self in the cognitive system. In this regard, Hommel (2018, see also Hommel, 2021) developed a promising approach in recent papers. He started from the Theory of Event Coding (TEC, Hommel et al., 2001) and assumes that the representation of the self and the representation of the others are event files consisting of a bundle of feature codes at a given moment (color, shape, location, but also motor properties and goals, etc.). In principle, the representation of the self (the minimal self) and the representation of the others do not differ, but the self has (1) preferential and, in part, exclusive access to our sensations (e.g., with regard to proprioceptive sensations). (2) The ideomotor principle as an integral part of TEC enables the planning and execution of motor activities and (3) the comparison between the predicted and actual motor outcomes allows us to judge fairly reliably whether we are the originator of an action or not. This lets us distinguish ourselves from the self of others.

Still, the event files of ourselves may also share features with the event files of others. A high degree of self-other overlap may promote mutual empathy, for instance (cf. Quintard et al., 2021). In the present context, such feature overlap is especially interesting when the other is an avatar. An increased self-avatar overlap is likely as the user sets the goals in the virtual environment, controls the avatar’s actions, and compares the predicted with the actual motion outcomes of the avatar. This makes the user feel as if she is the originator of avatar’s action, which might also lead to perceived body ownership. These are exactly the conditions that promote avatar-self merging.

The extent of self-avatar overlap is not fixed but varies with the user’s traits and features and with the avatar’s characteristics and action options. A user’s personality (Dunn and Guadagno, 2012) or gender and race (Dunn and Guadagno, 2019), for example, predict which avatar they choose. In turn, the appearance of the avatar influences the user’s behavior, and identification with the avatar increases with perceived interactivity (Hefner et al., 2007). Accordingly, and in contrast to other approaches, avatar-self merging describes a bi-directional process in which user and avatar influence each other. Furthermore, avatar-self merging is not seen as an all-or-nothing process but forms a continuum of varying intensities. Just as the extent of self-merging might be different between a plumber with their pliers and an arm amputee with their prosthesis, the difference is finally only gradual. Their tools, the pliers, and the prosthesis have become an integral part of their user’s lives, make their intentions and goals achievable, expand their action space, and make impossible actions possible. An avatar similarly increases the user’s action space and possibilities, but beyond that an avatar can be seen as a human(-like) being with its own appearance and character.

Successful avatar-self merging requires that the user puts themself in the situation of this character, that is, the user has to take its perspective. Perspective taking (PT) is an important process, when interacting with others. In its broader sense, it describes the ability to put oneself in the place of another person and to infer their mental states (e.g., percepts, feelings, beliefs, needs, and goals; Flavell et al., 1981; Steins and Wicklund, 1993; Birch et al., 2017). PT covers three mental aspects at least: affective PT (understanding another’s emotions and affects, i.e., compassion or empathy), cognitive PT (understanding [unobservable] processes within a person, e.g., this person is lying), and visual-spatial PT (considering the visual–spatial perspective of another person; cf. Steins and Wicklund, 1993).^2^ In the following, we discuss avatar-self merging against the background of affective, cognitive, and visual-spatial PT.

## Affective and Cognitive Perspective Taking: Adopting the Avatar’s Assigned Character

At first glance, acting with a self-controlled avatar in a virtual environment resembles a (social) situation in which a human observer attempts to infer the mental states of another person (here the avatar) in order to understand and predict its behavior. At second glance, as the avatar represents the user, the mental states of the avatar should be directly accessible to them—however, this does not mean that the assigned appearance and character of the avatar do not affect perspective taking.

Avatars are presented abstractly up to human-like. In some studies, avatars were found to be subjectively preferred, the more realistic they are (e.g., Fribourg et al., 2020). A more realistic avatar also seems to increase perceived body ownership (e.g., Latoschik et al., 2017), although this may not always be beneficial. Lugrin et al. (2015) reported that users feel stronger with a non-realistic but tough-looking avatar—a finding that is reflected in the so-called Proteus effect: Users adjust their behavior according to a randomly assigned appearance and/or character of an avatar. Yee and Bailenson (2007) showed that participants behaved in correspondence with stereotypes caused by the perception of their own avatar, for example, by being more confident when their avatar was taller. Similar effects have been demonstrated across different contexts, such as aggressive behavior (Ash, 2016), exercise habits (Fox and Bailenson, 2009), pro- and antisocial behavior (Yoon and Vargas, 2014), financial decisions (Hershfield et al., 2011), avatar’s age (Beaudoin et al., 2020; Reinhard et al., 2020), and many more (for an overview see Ratan et al., 2020). There is also evidence that users adapt not only their behavior but also their mental attitudes to the avatar (Banakou et al., 2013).

Current explanations of the Proteus effect do not refer to self-merging. For example, Peña et al. (2009) attributed the Proteus effect to priming and inhibition processes triggered by the appearance of the avatar. Their assumption is that an aggressive-looking avatar primes an aggressive model and inhibits the inconsistent non-aggressive one and that without assuming a recourse to self-merging processes. However, explanations like priming and inhibition on the one hand and self-merging on the other are not mutually exclusive. Priming and inhibition refer to the processes, while self-merging refers to whether and to what extent the user feels that the avatar belongs to them or not. Thus, avatar-self merging may be indicated, when the user adapts their behavior to the appearance and character of an avatar.

## Visual-Spatial Perspective Taking

**Figure 1:**
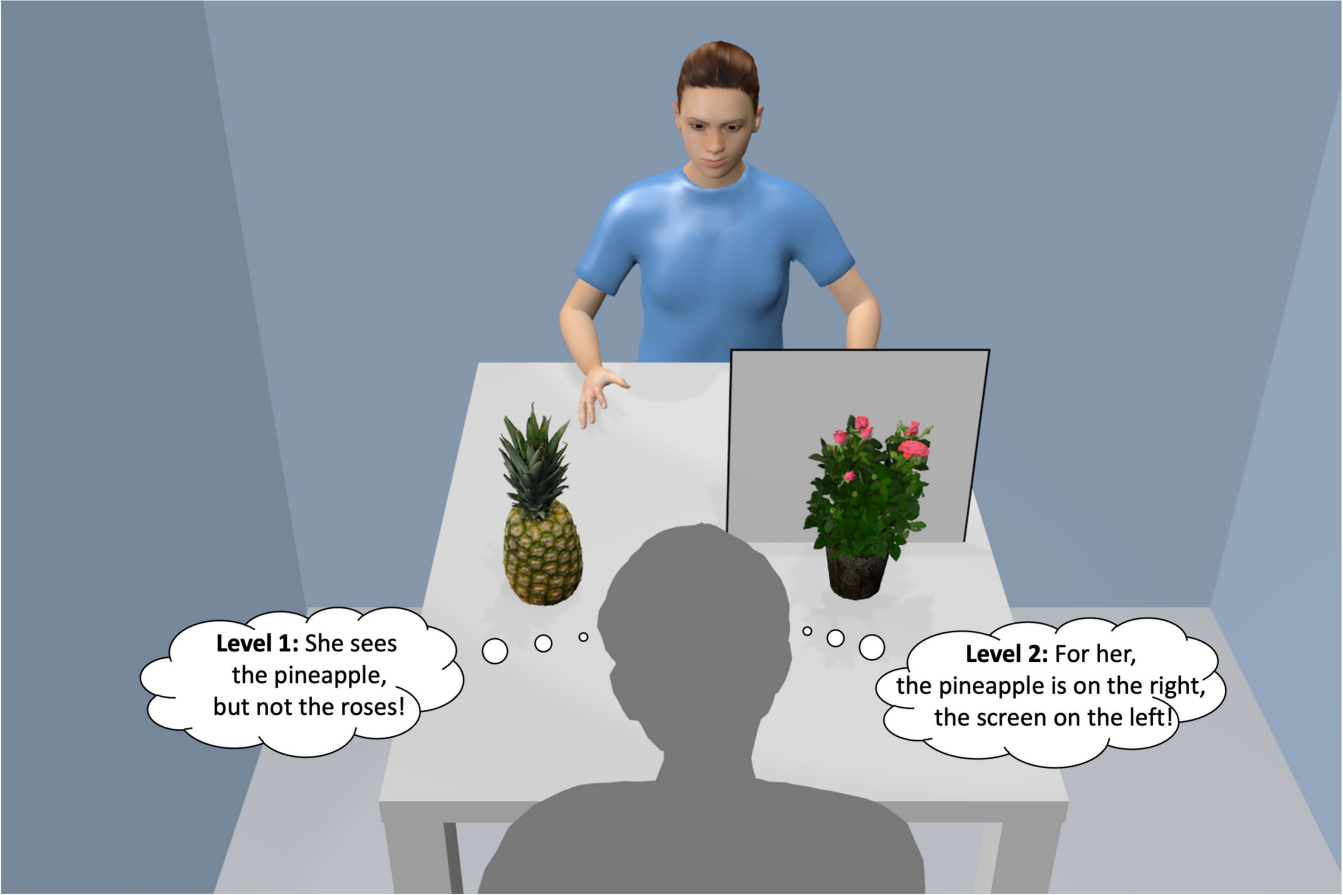
Level 1 and 2 visual-spatial perspective taking (VSPT) with regard to Flavell et al. (1981). [“Pineapple” (https://skfb.ly/6TQSO) and “Rose in a pot” (https://skfb.ly/6SDLR) by the sidekick are licensed under Creative Commons Attribution (http://creativecommons.org/licenses/by/4.0/)].

The dominant sense of humans is vision, and so it is not surprising that PT also covers the ability to see the space around another person from its perspective. This visual-spatial perspective taking (VSPT) accounts for what the other person (here the avatar) sees and how they see it (Flavell, 1977), for instance, whether objects are (partially) occluded from their view or whether they can see something that the observer (here the user) is unable to see. Research on VSPT has its origin in developmental psychology. Flavell et al. (1981) distinguished between two developmental levels of VSPT. While at the earlier “level 1 VSPT,” the child has insights into what objects are visible or occluded from the other’s point of view, “level 2 VSPT” adds further insights how others perceive the world, including deviating distances and deviating relative positioning from one’s own perspective (Figure 1). Level 2 VSPT is seen as a precondition for joint action planning with others and for solving social tasks from the other’s point of view (e.g., Freundlieb et al., 2017; Müsseler et al., 2019). Before getting into further details of level 2 VSPT, let is look at the different perspectives available for a user when dealing with an avatar in a virtual environment.

### The First and Third Person Visual Perspective

**Figure 2:**
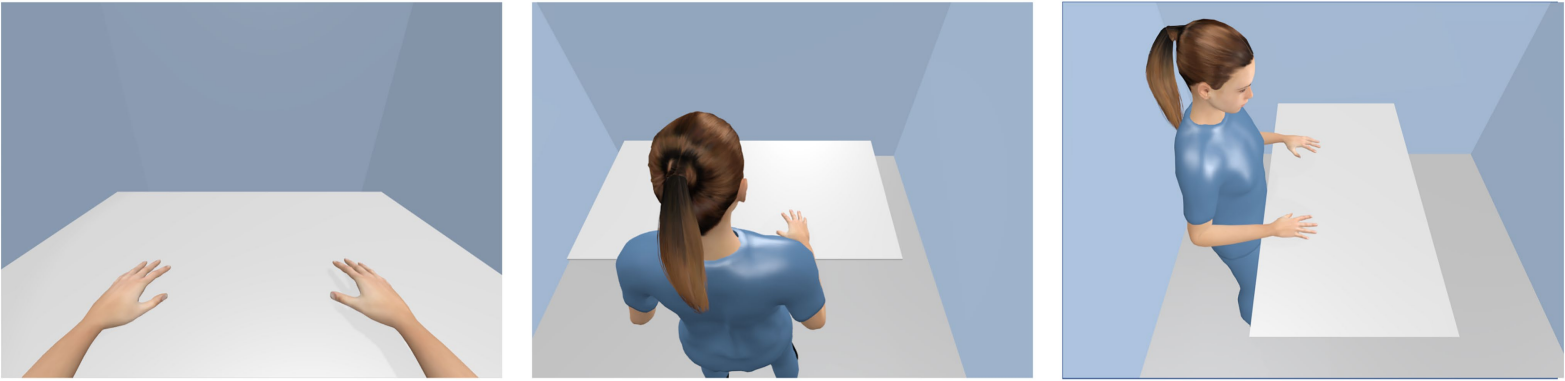
The first person’s visual perspective (**left panel**), the third person’s visual perspective (here slightly lateral from above, **middle panel**), and the rotated visual perspective (here 90° clockwise rotated from the user’s view**, right panel**).

The first person perspective is the view through the avatar’s eyes (Figure 2 left panel). The user sees the avatar’s arms and hands as possible effectors and can sometimes look down to the avatar’s legs (Pan and Steed, 2019), but the face, head, and back remain hidden (unless a mirror is in the virtual environment). Typical video games being played in the first person perspective are so-called first person shooters, such as Half-Life and the Call of Duty series. This perspective is often perceived as being close to reality, especially when the avatar’s hands are the acting effectors in that virtual environment.

In a recent study, Arend and Müsseler (2021) showed that the presentation of avatar hands in the first person perspective facilitated responding to affording objects compared to a condition in which no hands were presented. This effect may be related to the finding outside of virtual environments that visual-spatial attention is preferentially directed to objects close to our real hands (near-hand effect, cf. Reed et al., 2006; Colman et al., 2017; Agauas et al., 2020). If a user has successfully taken the avatar’s perspective and sees the avatar’s hands as their own hands, such effects should also be observable for the virtual hands, and this seems to be the case.

In the third person perspective, the user has the avatar’s body in view, while the viewing direction is roughly maintained. So, the avatar is shown from behind, above, and/or slightly lateral (Figure 2 middle panel).^3^ Typical video games being played in the third person’s perspective are Fortnite and the Witcher series.

Gorisse et al. (2017) carried out a study to compare the first with third person perspective. Their participants handled an avatar from either perspective in an immersive virtual environment. They found that the first person perspective enabled more accurate actions, while the third person perspective provides better spatial awareness (cf. the concept of self-location, Kilteni et al., 2012). Questionnaire data indicated the first person perspective as helpful to induce perceived ownership and to precise self-location. Kondo et al. (2018) also showed that the first person perspective was sufficient to induce perceived body ownership and that this impression was just as intense as the third person perspective with a whole-body avatar.

### The Rotated Visual Perspective

The rotated visual perspective is a special type of the third person perspective, in which a person observes another individual viewing a scene from a completely different angle (Figure 2 right panel). This situation characterizes primarily social encounters between humans, but it is also found in some video games with avatars (e.g., Grand Theft Auto 2 and games using isometric graphics or fixed camera positions).

**Figure 3:**
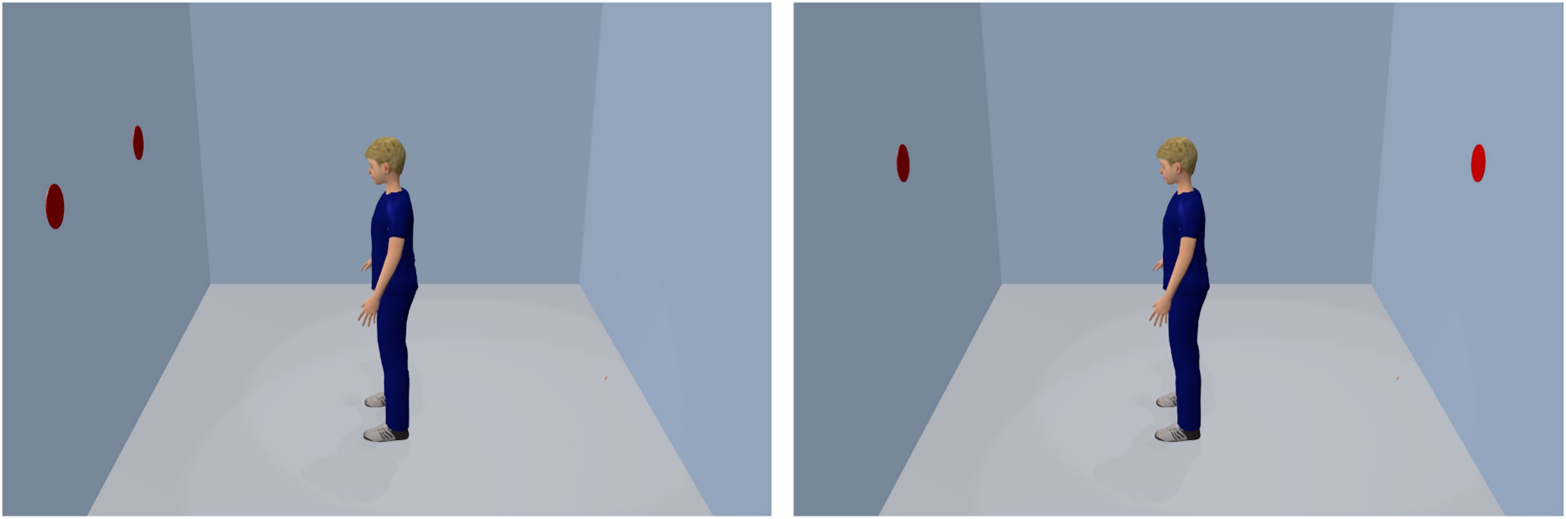
The dot-perspective task of Samson et al. (2010). Participants responded to the number of dots on the display. Reaction times are typically facilitated when the participant sees the same number of dots as the avatar (**left panel**), compared to when they see a different number (**right panel**).

Most of the research on VSPT has been conducted using this perspective, often with unanimated static avatars. An example is depicted in Figure 3, the so-called dot-perspective task introduced by Samson et al. (2010). The participant’s task was to respond to the number of dots on a display. Reaction times were found to be facilitated when the participant sees the same number of dots as the avatar (left panel), compared to when they see a different number (right panel). This finding was interpreted as evidence for spontaneous perspective taking and is probably related to the tendency of humans to align their direction of gaze with one another (Driver et al., 1999; Frischen et al., 2007; Kunde et al., 2011).

A problem for the present research question is that the dot-perspective task and its findings may account for perspective taking (including that of an avatar), but less likely for avatar-self merging. This is because this task is purely receptive in nature and does not require acting from an avatar’s perspective. We therefore favored the subsequent approach.

### The Rotated Visual Perspective and User’s Response Tendencies

The starting point for the following series of experiments was twofold (cf. Müsseler et al., 2019; Böffel and Müsseler, 2019b): First, a rotated visual perspective has the consequence that the spatial relations in a scene are different from the avatar’s point of view and from the user’s point of view. Second, cognitive psychology has shown that humans do possess predetermined response tendencies toward objects in space that sometimes facilitate one response and impede the other. The response tendencies of interest here are summarized under the label of spatial stimulus-response compatibility (for an overview see, e.g., Proctor and Vu, 2006). A typical finding in compatibility experiments is, for example, that a left (right) stimulus is responded faster and less error-prone with a compatible left (right) response than with an incompatible right (left) response.

In the present context, our aim was to confront participants with a situation that contained conflicting response tendencies from their own and their avatars’ points of view and to observe which of the response tendencies dominated. If a user can become one with an avatar and act as if they are the avatar, the response tendency from the avatar’s point of view should prevail and override the one from the user’s point of view.

#### The Avatar-Compatibility Task

**Figure 4:**
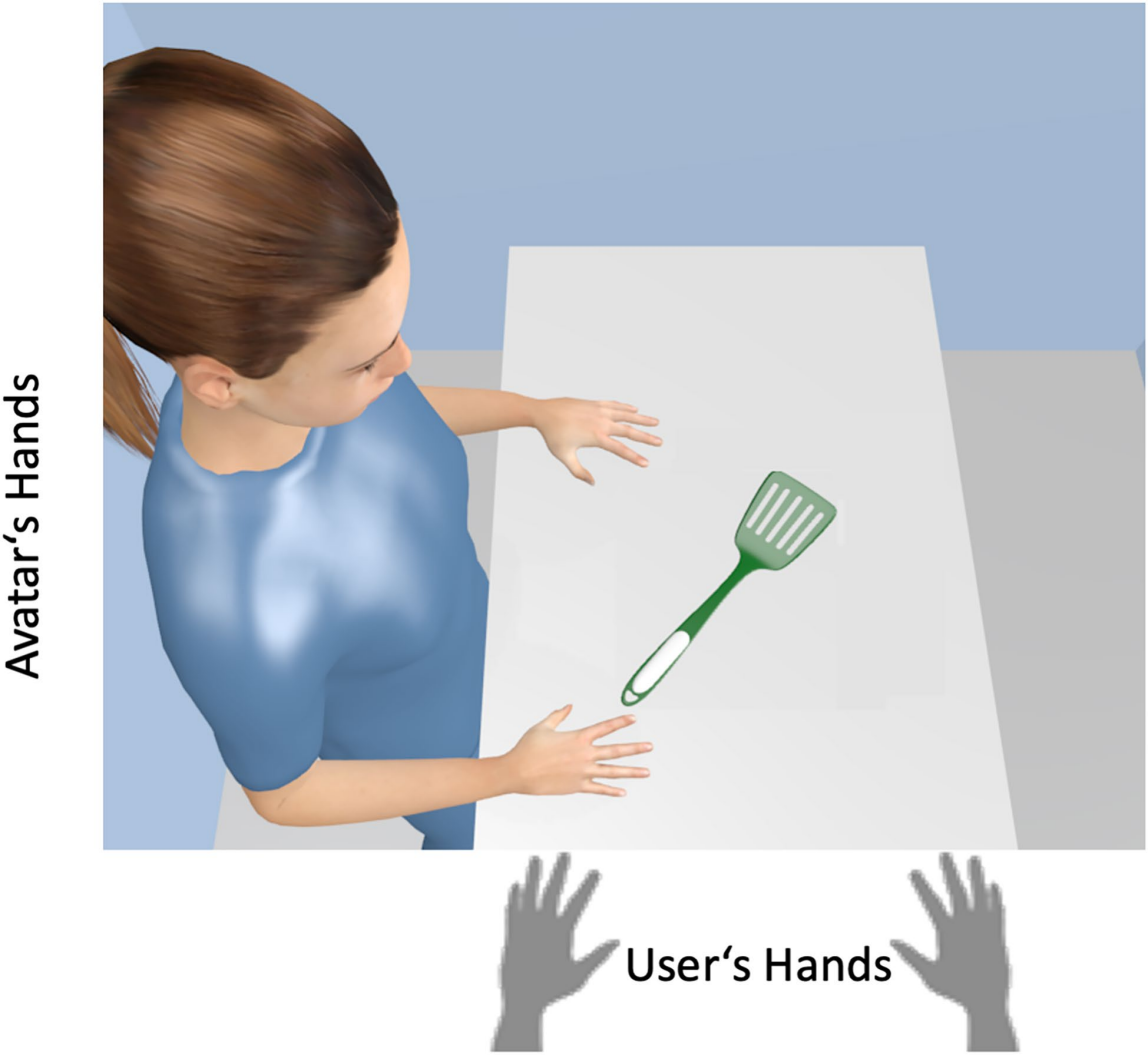
The principle of the avatar-compatibility task. A user controls the left and right hand of an avatar with left and right keystrokes. If the avatar is to grasp the handle of the pan lifter, a right response from the avatar’s point of view should be preferred (which required a right response of the user). However, the handle is oriented to the left from the user’s point of view, which should facilitate a left response. Only if the user takes the perspective of the avatar, the right response should have the advantage. Our findings support consistently this assumption. [“Spatula” (https://skfb.ly/6QWQs) by Matthew is licensed under Creative Commons Attribution (http://creativecommons.org/licenses/by/4.0/). The color of the pan lifter was adjusted].

Consider the following situation: A user controls the left and right hand of an avatar with left and right keystrokes. If the avatar is to grasp the handle of a pan lifter as shown in Figure 4, this suggests a right response from the avatar’s point of view. However, the handle is oriented to the left from user’s point of view, which should facilitate a left response. Thus, user’s and avatar’s perspective suggest different response tendencies and only if the user takes the perspective of the avatar, the right response should have an advantage. Or in other words, we hypothesized that users should neglect their own perspective when they become one with the avatar.

This was what we found in several studies and we refer to this compatibility effect from the avatar’s point of view as the avatar-compatibility effect. In the experiments of Müsseler et al. (2019; see also Böffel and Müsseler, 2020a), participants should take the perspective of a rotated avatar and pressed ipsilateral or contralateral left-right keys in response to lateralized colored disks. We found consistently that compatibility effects were tied to the avatar’s view but not to the participant’s view. In other words, participants were able to perform compatible ipsilateral responses from the avatar’s point of view faster and less error-prone than incompatible contralateral responses, even though from the participant’s point of view the compatibility relationships were reversed. We interpret this finding as evidence that participants are able to implement their behavioral tendencies into the avatar, thereby neglecting their own perspective. Further note that compatibility findings (i.e., without an avatar) are usually very robust and can hardly be eliminated even by practice. It is therefore astonishing that the mere instruction to take the perspective of the avatar was able to turn the results into the opposite.

Böffel and Müsseler (2018) extended the finding by varying the degree of induced body ownership of the avatar *via* instruction. Half of the participants were informed to have complete control over an avatar (high-ownership condition), while the other half of the participants were informed that the avatar has its own will (low-ownership condition). Although the events on the screen were exactly the same in both conditions (for details of the experimental procedure, see Böffel and Müsseler, 2018), the results showed that the avatar-compatibility effect was more pronounced in the high-ownership condition than in the low-ownership condition. We attributed this to an increased avatar-self merging in the high-ownership condition compared with the low-ownership condition. This conclusion was supported by questionnaire data showing an increased body-ownership score in the high-ownership condition than in the low-ownership condition. The study demonstrated that body ownership and avatar-self merging rely on a person’s interpretation of a situation that can be induced by the instruction.

#### The Avatar-Simon Task

**Figure 5:**
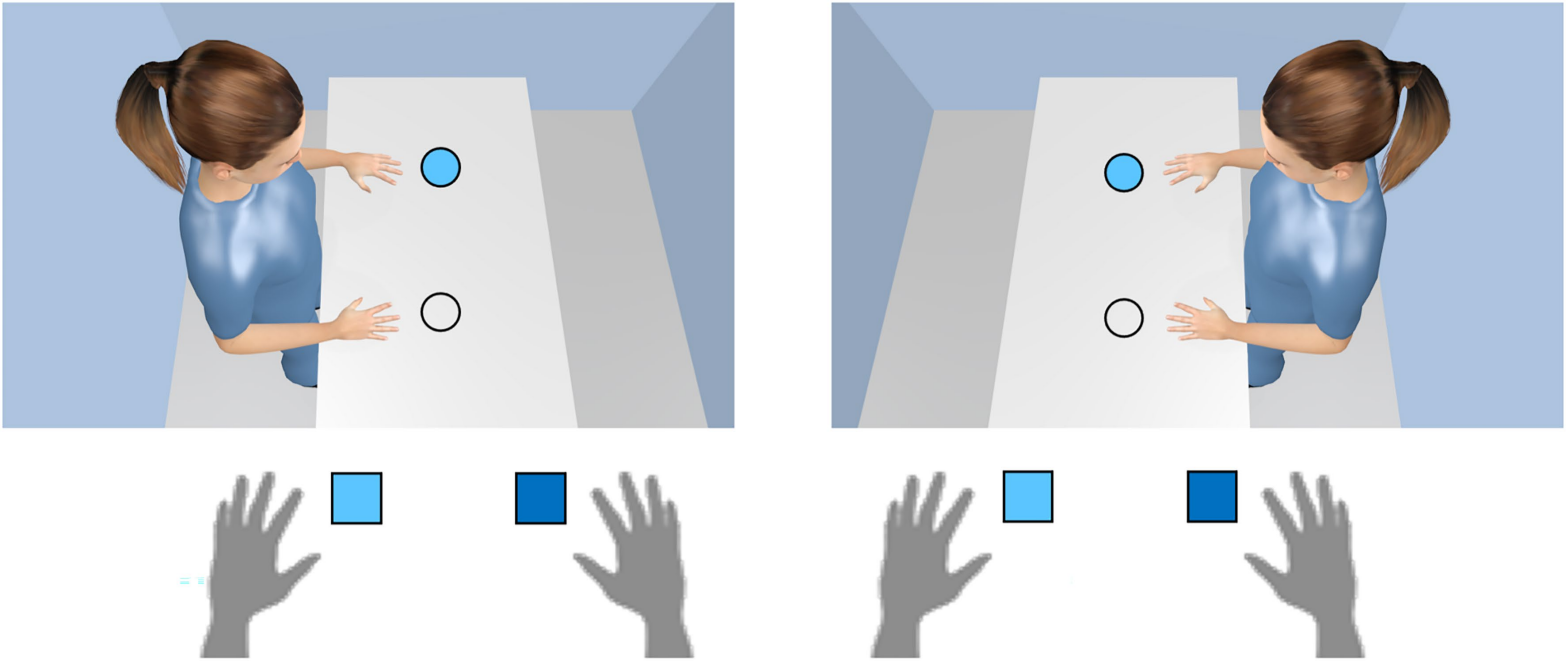
The principle of the avatar-Simon task. Participant’s task is to press on a light (dark) blue disk a left (right) key (here with light blue disk only). Disk positions are randomly assigned to the upper and lower position (here the upper position only). In the left panel, a left response is required, which corresponds to the avatar’s left hand. In the right panel, a left response is also required, but it does not correspondent with the avatar’s left hand, but its right hand. As a result, reaction times and fewer errors are observed with the avatar on the left side than with the avatar on the right side.

While in the two previously mentioned studies the avatar could not be ignored to solve the task successfully, there is also evidence that the avatar’s point of view is even adopted when it is in principle irrelevant for the task. A compatibility effect without an avatar, but task-irrelevant spatial positions is observed in the so-called Simon task (for an overview, see Hommel, 2011). Here, participants respond with the left-hand key to one color, for example, and with the right-hand key to another color that is presented on the left or right side of a display. Although stimulus position is task-irrelevant, spatially compatible conditions (e.g., left stimulus and left response) produce faster responses and fewer errors than spatially incompatible conditions (e.g., left stimulus and right response). Recent studies in our lab demonstrated that the Simon effect can also be observed when an avatar is added to the scene (Figure 5; Böffel and Müsseler, 2019b; von Salm-Hoogstraeten et al., 2020). By rotating the stimulus positions and the avatar by ±90° from the user’s point of view, the stimulus does not contain spatial information on the left-right dimension from the user perspective, but only from the avatar perspective.

The results of the experiments indicated that actors take the avatar’s perspective since they reacted in accordance with the Simon effect from the avatar’s perspective (avatar-Simon effect; Böffel and Müsseler, 2019a,b, 2020b; von Salm-Hoogstraeten et al., 2020; von Salm-Hoogstraeten and Müsseler, 2021b). This finding also occurs spontaneously, that is, it is observed even when the participant is not instructed to take the avatar’s perspective. However, when the avatar was replaced by a disk or an arc, the avatar-Simon effect disappeared (Böffel and Müsseler, 2019b). It is therefore obvious that not any simple object can trigger the effect and that a human-like character is beneficial. We will come back to this point below.

While the standard Simon effect (i.e., without an avatar) demonstrates that participants cannot ignore the position of a stimulus, the avatar-Simon effect shows additionally that they apparently cannot ignore also a (virtual) reference person either (for compatibility studies in social situations with human reference persons, see also Freundlieb et al., 2016, 2017).

### “Seeing” the Avatar’s Perspective vs. Referential Coding

**Figure 6:**
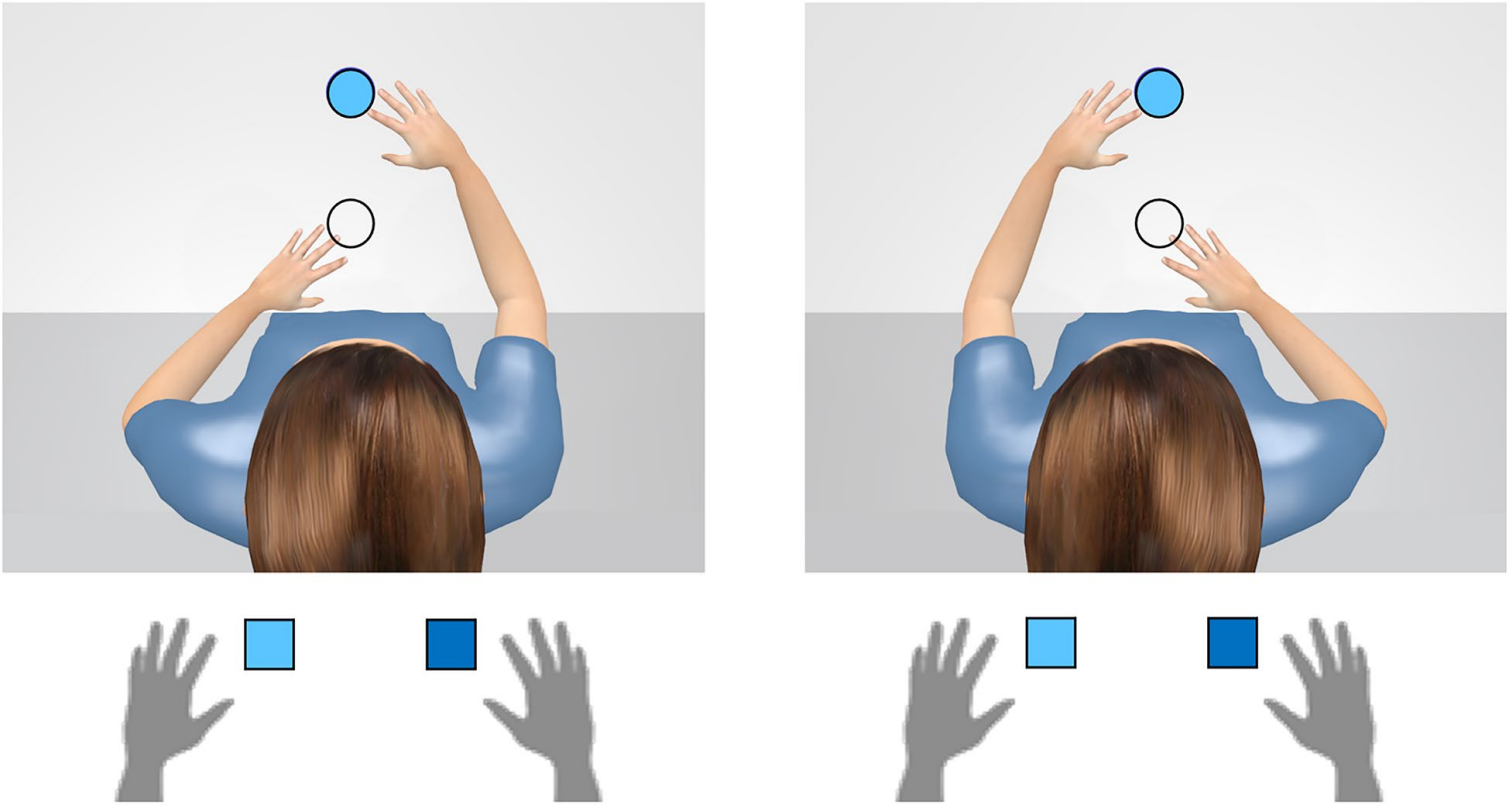
The second scenario in the study of von Salm-Hoogstraeten et al. (2020). Participant took the first person perspective of the avatar and the avatar’s right and left hand were now at the upper and lower stimulus position. The results showed pronounced avatar-Simon effects depending on the hand position of the avatar.

Visual-spatial perspective taking is often understood as a process based on a visual–spatial representation created from another person’s point of view. If the participants take the view of the avatar, they literally “see” the objects on the left or right side (e.g., Flavell, 1977; Costantini et al., 2011; Ward et al., 2019; for a critique of this view see Cole and Millett, 2019). Recent studies from our lab cast doubt on this simplification of the perspective-taking mechanism. von Salm-Hoogstraeten et al. (2020) compared two avatar scenarios: The first scenario was similar to the one illustrated in Figure 5. An avatar sat either to the left or to the right of a table and participants performed a Simon color-classification task to left-right stimuli from the viewpoint of the avatar. Note, that from the participants’ point of view, the stimuli were arranged one above the other (i.e., with no spatial information on the horizontal dimension). The second scenario is illustrated in Figure 6. The participant took the first person perspective of the avatar and the avatar’s right and left hand were now at the upper and lower stimulus position. In this scenario, only the avatar’s hands formed the left and right relation to the stimulus positions. A perspective-created visual representation could only account for effects in the first scenario while the avatars’ hands could produce a left-right frame of reference in both scenarios. The results showed pronounced avatar-Simon effects in both scenarios.

We interpreted this finding as evidence for the view that the avatar’s position, and also the spatial positions of any other object in the scene, could be selected as a new spatial reference point from which the spatial relationships of the objects to each other could be redefined. That spatial coding of objects could arise in reference to other objects is an idea postulated by the referential coding account that was originally proposed to explain spatial compatibility effects in the standard Simon task (Hommel, 1993), and then was applied to the orthogonal compatibility task (Lippa, 1996; Cho and Proctor, 2005) and the object-based Simon task (Cho and Proctor, 2010; Arend and Müsseler, 2021). Recently, the referential coding account was also extended with regard to the joint Simon task (e.g., Dolk et al., 2013).

According to the referential coding account of perspective taking, the basic spatial map develop from the user’s perspective, which, however, already contains all spatial relationships between objects in the visual space (cf. the visual sensory map of van der Heijden et al., 1999). Consequently, the user does not need to create a new visual-spatial map from the avatar’s perspective but rather recodes the existing coordinates with regard to the new reference point. Thus, there may be little visual in visual perspective taking.

Generally, the recoding of objects within a new spatial reference frame is mostly investigated in terms of stimulus-coding, the mental representation of the objects and their positions. In a recent study (Böffel et al., 2020), we modified the avatar-Simon task by using centrally presented numbers as targets in order to remove the spatial variation of the stimuli. In these experiments, recoding the stimulus position could not be responsible for compatibility since the stimulus did not change its position. However, the avatar’s movements could be recoded within the spatial reference frame and we still observed a compatibility effect, demonstrating that not only stimuli but also action effects are recoded from the avatar’s point of view (Böffel et al., 2020). Therefore, the role of action effects and their spatial coding and interpretation seems to be crucial for avatar-based compatibility and was the topic of a series of further experiments.

## Visual Perspective Taking When Controlling Avatar’s Movements

While the studies in the prior section used an avatar from whose perspective the user was supposed to act, the avatar itself did not perform the corresponding actions in all studies (e.g., not in Müsseler et al., 2019 and von Salm-Hoogstraeten et al., 2020).^4^ It seems to be enough to imagine these movements (as in tool use, cf. Müsseler et al., 2014). However, it is indisputable that user movements that are synchronously and consistently mirrored in corresponding avatar movements increase perceived ownership and agency (e.g., Sanchez-Vives et al., 2010; Kilteni et al., 2012; Fox et al., 2015; Pfister et al., 2017; Kondo et al., 2018). The reason for this has already been noted in the Introduction: The ideomotor principle, as an integral part of the event-coding approach, allows to transform anticipated actions into executed actions (cf. James, 1890; Hommel et al., 2001; Kunde et al., 2004; Shin et al., 2010; Pfister, 2019). Furthermore, the comparison between anticipated and experienced outcomes contributes to who feels ownership of an action. Note that realizing these relationships is not a given from birth but is acquired in a developmental process in early childhood (e.g., Elsner and Adam, 2021). It also does not matter much where the action effects occur. In other words, whether action effects are anticipated in the proximal action space of the user (e.g., as tactile sensations at their hand) or in the distal space when a lamp is switched on or in the distal virtual space of the avatar depends alone on the user’s intentions (cf. the findings with regard to tool use, e.g., Sutter et al., 2013).

Böffel and Müsseler (2019a) varied the participants’ control over their avatar using the avatar-Simon task. In a full-control condition, the avatar consistently moved the left-right hand with the corresponding left-right keypress of the participant. In a less-control condition, the avatar moved a random hand instead, making the distal hand movements impossible to predict and effectively useless for action planning. The results confirmed our hypothesis that high control resulted in higher perceived body ownership and an increased avatar-Simon effect, providing evidence of increased avatar-self merging in both self-report and behavioral data (see also Ma and Hommel, 2015).

Consistent action effects at the avatar also allow the user to differentiate their avatar from other characters (which are controlled by another user or by the computer program). Self-other distinction is an important requirement for successful interactions in real and virtual environments (e.g., Mattan et al., 2016). Only the identification of one’s own avatar and the differentiation from others enables successful action. This can be achieved by consistent feedback of the anticipated action effects at the own avatar. von Salm-Hoogstraeten and Müsseler (2021b) showed that users preferred to take the perspective of the avatar that consistently mirrored their actions, even though another virtual character took a similar perspective. The study also showed that perspective taking is not that spontaneous, as sometimes assumed (cf. Samson et al., 2010; Freundlieb et al., 2016, 2017). Instead, perspective taking is likely to benefit from action-based and thereby top-down controlled processes.

Besides the consistency of action effects, the synchronicity and movement correspondence of action effects of the avatar is likely to be conducive to avatar-self merging. Although not examined in a study with an avatar, it is likely that the actor no longer experiences themselves as the originator of an action, when the action effect is presented too early (e.g., before the user’s action) or too late (cf. Haering and Kiesel, 2015; Dignath and Janczyk, 2017). Similarly, performance decreases if action effects are durationally or spatially not in correspondence with the participant’s movements, e.g., when a short keystroke is transferred into a long keystroke or a right movement into a left movement (or vice versa; Pfister et al., 2017; Liesner et al., 2020).

As with the rubber-hand illusion, attention should also be paid to corresponding hand-hand postures (cf. Costantini and Haggard, 2007). In yet unpublished experiments in our lab, we were able to show that both the avatar-compatibility effect and the avatar-Simon effect disappeared when either the avatar or the user crossed their hands. This was despite the fact that hand-hand correspondence still applied, that is, a left (right) button press resulted in a left (right) action effect at the corresponding hand of the avatar. Only when both pairs of hands, the user’s and the avatar’s, were crossed, the effects re-appeared in both objective and subjective measures (Müsseler, 2019). In summary, appropriate action effects at the avatar (with regard to consistency, synchronicity, correspondence, and posture) not only facilitate self-merging with the avatar, they also contribute essentially to self-other distinction within the virtual environment.

## Visual-Spatial Perspective Taking as a Social Ability

There is an ongoing debate about whether the ability of VSPT emerges exclusively in social interpersonal contexts (referring to the more cognitively demanding level 2 VSPT; Flavell et al., 1981). Can one also take the perspective of a (humanoid) character or even an object? Since the seminal paper of Shepard and Metzler (1971), the ability to mentally rotate an object is undisputed. However, note that in VSPT, humans perform a mental self-rotation in order to take the perspective of others. This makes perspective taking with (humanoid) characters and mental rotation with objects dissociable (e.g., Zacks and Michelon, 2005; Kessler and Thomson, 2010). Still, Hegarty and Waller (2004) reported that both abilities are highly correlated, which could indicate that perspective taking is not tied to human or humanoid characters. Accordingly, we observed the avatar-Simon effect also with a headless robot that could hardly be described as humanoid (von Salm-Hoogstraeten and Müsseler, 2021a). However, the robot had two arms and perhaps that was enough to yield a humanoid appearance. At least the two arms could have specified the direction of perspective taking, which is normally determined by the gaze direction or head orientation of the observed character. This in turn strengthens the social view of perspective taking, because objects usually do not have this orientation.

Evidence emphasizing the social aspect of VSPT has been recently reported in a study by Ward et al. (2019). Their participants judged normal or mirrored letters (e.g., an R or an Я) shown with various rotation angles on a flat table. Either only the table was presented or an avatar sat to its left or right or a lamp directed toward the letters was placed at the same position as the avatar. The authors observed lower response times with low rotation angles of the participants to the letters compared to larger angles. However, lower response times were also found when the rotation angles were low with regard to the avatar, although, then, the angle with regard to the participants was high. Most importantly in the present context, no such effects were observed with the lamp presented instead of the avatar. This is in line with our observations that the avatar-Simon effect disappeared when a disk or an arc was presented instead of the avatar (Böffel and Müsseler, 2019b).

To a last example focusing on the social aspect in virtual environments: In the experiments of Bönsch et al. (2018, 2020), users controlled an avatar in space in the first person perspective, which was approached by either a happy-looking or angry-looking virtual character. Users preferred to be at a greater distance from or walk past the angry-looking character than the happy-looking character. These results show that the regularities that apply in human-human interaction are also adopted in virtual environments. Whether this can be interpreted beyond doubt as evidence for avatar-self merging is debatable, but at least maintaining these regularities in virtual environments should facilitate it.

## Conclusion

In this paper, we started with the event-coding approach of the self (Hommel, 2018, 2021) and showed that self-avatar overlap is predestined to give rise to avatar-self merging, mainly due to the transfer of the user’s motor activities into corresponding avatar activities. For successful avatar-self merging, it seems essential to us that the virtual environment opens up possible actions for the user to realize their intentions. Whether action control is achieved in a real environment or an artificial one is not decisive for the self.

In our experiments, users were confronted with situations that contained conflicting response tendencies from their own and their avatars’ points of view. The results revealed that users often overrode their own response tendencies and acted as if they were the avatar. As a rule, this observation was accompanied by increased scores in perceived ownership and agency (Böffel and Müsseler, 2018, 2019a), suggesting avatar-self merging. The procedure of our experiments could be applied to a variety of other response tendencies that are known in cognitive psychology.

For example, so far, we have dealt almost exclusively with spatial stimulus-response compatibilities, that is, both stimuli and responses exhibited a critical spatial position (but see Böffel et al., 2020). However, there are also stimuli that trigger response tendencies regardless of their spatial position. For instance, the presentation of a baby photo usually produces an approach behavior, whereas the photo of a violent scene produces an avoidance behavior (e.g., gathered with a speeded joystick response, Eder et al., 2012). If an avatar is added to the scene, from whose point of view the photos are to be judged, the experimenter can again create a discrepancy from the user and avatar point of view and examine which response tendency dominates. Further, it would be intriguing to examine whether the user also adopts social attitudes of an avatar, which are associated with its ethnicity, its gender, or—more general—its group affiliation. Again, to clearly interpret the results, it would be important to ensure an experimental setup with a discrepancy between the user’s attitudes and the avatar’s affiliation.

Following the event-coding approach, avatar-self merging is not seen as an all-or-nothing process, but rather as a process to which different features may or may not contribute. As various studies have shown, the human information-processing system is flexible enough to adapt its behavior not only to various real-world environments but also to novel artificial virtual ones. As a prerequisite for avatar-self merging, we consider the user’s ability to successfully take the perspective of an avatar in affective, cognitive, and visual–spatial terms. However, this is not to say that these factors are adopted in their entirety. This remains an empirical question.

In addition to the cognitive aspects, the extent of avatar-self merging is of course also determined by the technical implementations of the virtual environment. The more immersive a virtual environment is, the more likely our senses are to experience an environment as “real,” and the more pronounced avatar-self merging is likely to be. However, immersion also means that the senses important for action planning and action execution are implemented, that is, the efferent mechanisms triggering an action and the afferent mechanisms controlling them. In this context, it should also be pointed out that most (action) events in our natural environment can be experienced in a multisensorial manner (i.e., visual, auditory, tactile, and/or proprioceptive). This is often missing in the virtual applications.

Even if we succeeded in realizing all these components in an immersive environment, the problem of sensorimotor transformation would remain. It consists in transforming a proximal movement (e.g., a user’s keypress) into a non-corresponding distal movement (e.g., a movement of the entire hand including the arm of an avatar; cf. this problem in tool use, Sutter et al., 2013). Thus, this transformation rarely follows a 1:1 rule but is, for example, longer or shorter, amplified, or reduced in force, and this not necessarily in a linear manner. Acquisition and execution of distal movements in the presence of sensorimotor transformations are challenging for any user. That is the bad news. The good news is that the human users have the ability to acquire these transformations (although sometimes with a lot of practice) and then can act accordingly. As a consequence, avatar-self merging needs time and occurs only when the users have sufficiently internalized the transformation rule between proximal and distal action effects.

## Author Contributions

JM: conceptualization, writing—original draft, funding acquisition, project administration, and supervision. SS-H: conceptualization, visualization, and writing—review and editing. CB: conceptualization and writing—review and editing. All authors contributed to the article and approved the submitted version.

## Funding

This study was supported by the Deutsche Forschungsgemeinschaft (DFG, German Research Foundation; project number MU 1298/11-1) and was associated with the DFG Priority Program “The Active Self” (DFG SPP 2134).

## Conflict of Interest

The authors declare that the research was conducted in the absence of any commercial or financial relationships that could be construed as a potential conflict of interest.

## Publisher’s Note

All claims expressed in this article are solely those of the authors and do not necessarily represent those of their affiliated organizations, or those of the publisher, the editors and the reviewers. Any product that may be evaluated in this article, or claim that may be made by its manufacturer, is not guaranteed or endorsed by the publisher.

## Footnotes

^1^Other authors (e.g., Fribourg et al., 2020; Peck and Gonzalez-Franco, 2021) describe this feeling as avatar embodiment. However, in cognitive psychology, the term embodiment is used completely independent from artificial characters, instead it refers to the (theoretical) view that considers body states and actions as important or obligatory components of cognitive processes (e.g., Wilson, 2002). This was another reason not to use the term embodiment in the present context.

^2^The ability of PT is inseparable from the so-called Theory of Mind (ToM, cf. Premack and Woodruff, 1978; Baron-Cohen et al., 1985). Both terms are sometimes used interchangeably (e.g., Birch et al., 2017), other researchers use a more complex ToM to emphasize the observer’s insight that persons being observed may be in an individual state that differ from those of others. An observer, so to speak, can develop different ideas about what might be going on in the other person and weighs these ideas against each other in order to understand and to response accordingly (e.g., Harwood and Farrar, 2006). This comprehension of ToM, the possible weighing of different mental states, contributes only little to the present research question and is therefore neglected here.

^3^Originally, labeling as first, second, and third person perspective comes from linguistic. First person is the I/we perspective, second person is the you perspective, and third person is the he/she/it/they perspective. However, the distinction of the second and third person perspectives does not make sense when considering the spatial relationships.

^4^In these studies, a static unanimated avatar was used to clearly attribute the findings to perspective taking and not to the appearance of anticipated action effects at the avatar (see below and Kunde, 2001; Müsseler and Skottke, 2011).
